# Books and Authors

**Published:** 1910-06

**Authors:** 


### BOOKS AND AUTHORS.

**Report of the Commissioner of Education** for the year ended June 30, 1909. Vols. 1 and 2. Washington. Government Printing Office.

These books are replete with statistics and other information to all interested in advancing education. From it we learn items of interest to the medical profession in particular. In the table for 1908-9 are 144 medical schools, five less than in that of the preceding year. The number of medical students enrolled was 22,158, a total of 629 less than in the previous year. This loss was chiefly among the standard medical schools,—regular,—though the homeopathic schools lost 37 students. On the other hand the eclectic and physiomedical schools were increased by 126 students. Various reasons are offered by way of accounting for a decrease of 4,904 medical students in the last six years, two of which seem sufficient to meet the largest part of the decrease. First, the increased rigor of the examinations by medical boards; and, second, increased cost of attendance upon a four-year course.

Several other questions of importance are discussed in the report, such as preventive medicine, foreign medical students in the United States, the Rockefeller gift for exterminating the hookworm, and some lesser topics.

**A Practical Treatise on Ophthalmology.** By **L. Webster Fox,** M.D., Professor of Ophthalmology in the Medico-Chirurgical College, Philadelphia. Octavo, pp. 832. Illustrated with 6 colored plates and 300 illustrations in the text. New York and London: D. Appleton & Co. 1910. (Cloth, $6.00.)

Ophthalmologists who teach or practise, one or both, will welcome this work, as it presents the subject in detail and in all its modern aspects. Fox has been recognised for a long time as one of the foremost ophthalmologists in the country, a teacher of renown, and a man of great experience in diseases of the eye.

This treatise represents the status of ophthalmology today, offered in a comprehensive form, giving not only the author's own experience, but also that of his colleagues throughout the world who have contributed to the science material of value. It therefore becomes a work for the student, the practitioner, the teacher, and the specialist. To all these it appeals as a safe guide in the diseases with which it deals.

Short chapters first are given on the development, the anatomy and the external examination of the eye. Then comes a comprehensive chapter on diseases of the eyelids, which every practitioner should be prepared to deal with. The lachrymal apparatus, the conjunctiva, the cornea, and the sclera, these are presented in rapid succession and their diseases handled with skill.

Passing along we soon find a masterful chapter on diseases of the retina which will arrest the attention of the specialist. Under diseases of the optic nerve we find much of interest. Fox says the term amaurosis, formerly applied to partial or complete blindness, is rapidly falling into disuse and is being replaced by the term amblyopia, both terms indicating differences in degree only. He gives an ingenious test for malingering,—the word Friend painted upon glass on a black back ground in green and red letters alternately placed. The method of the employment of this device is described in detail and it should be known to everyone who examines eyes for the public services, or for institutions and other occupations where malingering may be practised. The tests for color blindness also are set forth in an instructive manner, Williams's lantern test being described and illustrated. The chapter on glaucoma is interesting in a special degree, giving its classification, diagnosis, and treatment in a most satisfactory manner. Refraction always possesses special interest, and Fox makes it particularly so in a chapter of eighty pages, that is clear in its diction and concise in its statements. A chapter on general operative technic and one on laboratory technic bring the book to a close. We regard the work as the best general treatise on ophthalmology that has appeared in recent years.

---

**Textbook of Medical and Pharmaceutical Chemistry.** By **Elias H. Bartley,** B.S., M.D., Ph.G., Professor of Chemistry, Toxicology and Pediatrics in Long Island College Hospital. Seventh edition. 12 mo, pp. 734. Illustrated. Philadelphia: P. Blakiston's Son & Co. 1909. (Cloth, $3.00 net.)

Bartley's chemistry has become a standard authority both with teachers and pupils. Four years have intervened between the sixth and seventh editions, yet comparatively but few changes have been found necessary. The text has been revised, typographical errors and misleading statements have been eliminated or corrected. This author is a teacher of experience, well qualified to judge of the requirements of students, and has made a book that meets their needs in a most satisfactory manner.

---

**Examination of the Urine:** A Manual for Students and Practitioners. By **G. A. DeSantos Saxe,** M.D., Instructor in Genitourinary Surgery, New York Post-Graduate Medical School and Hospital. Second edition. 12 mo, of 448 pages, illustrated. Philadelphia and London: W. B. Saunders Company. 1909. (Cloth, $1.75 net.)

This book deals with one of the most important topics in clinical diagnosis which is presented to the physician. Every clinician must know how to interpret the manifestations of the kidney excretion in health and disease, and to place its various evidences at their true value.

At the close of each chapter, Saxe groups a number of questions that relate to the topic dealt with, which serves to fix the tests and other material in the mind. This new edition has been made to embrace all the later points pertaining to urinary examination, while the tests that have proved valueless have been dropped. It is one of the better guides to urinary examination extant.

**Spondylotherapy:** Spinal Concussion and the Application of Other Methods to the Spine in the Treatment of Disease. By **Albert Abrams**, A.M., M.D. (University of Heidelberg), F.R.M.S. (Consulting Physician to the Mount Zion and French Hospitals, San Francisco; formerly Professor of Pathology and Director of the Medical Clinic, Cooper Medical College, San Francisco. Octavo, 417 pages, with 97 illustrations. San Francisco. The Philipolis Press. (Cloth, $3.50.)

This author presents a book that contains much of interest and value relating to spinal therapeutics. He makes an attempt to unfold the occult or mysterious claims of the chiropractic system and other similar methods. The chapters relating to visceral disease and the one dealing with pain,—the latter the final chapter in the book,—are of considerable interest. As we have already intimated it contains much of good, but also much of the speculative or "not proven" order. We fail to find much in it that contributes to medical science or progress.

---

**Nutrition and Dietetics.** By **Winfield S. Hall**, Ph.D., M.D., Professor of Physiology in Northwestern University Medical School, Chicago. Small octavo, pp. 315. New York and London. D. Appleton & Co. 1910. (Cloth, $2.00.)

The increasing knowledge pertaining to the part nutrition plays in the maintenance of health, makes its study one of the most interesting and profitable of the laboratory series. Professor Hall is a physiologist of experience and a teacher of judgment, hence what he says possesses great interest. His book is the product of ten year's work in the laboratory and in the lecture room, based upon latest researches in animal nutrition. It is a concise textbook intended for undergraduates and nurses in particular, but is adapted also to the requirements of the general practitioner. The author has drawn on contemporary resources here and there, when needed to elaborate or accentuate a fact or facts, and has offered a volume that attracts through its material and the manner of its presentation.

---

**Modern Surgery.** General and Operative. By **John Chalmers DaCosta,** M.D., Professor of Surgery and Clinical Surgery in Jefferson Medical College, Philadelphia. Sixth edition, revised and enlarged. Octavo, pp. 1502. With 966 illustrations, some in colors. Philadelphia and London. W. B. Saunders Co. 1910. (Cloth, $5.50.)

That DaCosta has become an accepted authority on the surgery of today is apparent from the fact that six editions of his work have been required since it was first published in the autumn of 1894. It is three years since the fifth edition was printed; meanwhile, many things have occurred, some of value, some not; but the author appears to have sifted very carefully, taking pains to record those that are of use, or that contribute to the improvement of surgical science. There are so many of this class, that great change has been made in this issue, requiring alterations or additions to nearly every section. It is pertinent, therefore, to say that this sixth edition represents the surgery of 1910. Da Costa has incorporated, besides his own, the work of every prominent surgeon of the period.

---

**The Elements of the Science of Nutrition.** By **Graham Lusk,** Ph.D., M.A., F.R.S. (Edin.), Professor of Physiology at Cornell Medical School, New York. Second edition, revised. Octavo of 402 pages, illustrated. Philadelphia and London: W. B. Saunders Company, 1910. (Cloth, $3.00 net.)

This work first appeared in 1906, and this edition presents those facts that have been observed since that time, besides those the book first embodied. Nutrition is so important that anything that contributes to a better understanding of its mysterious processes, is welcome to the alert physician. Lusk is an investigator of accomplishment, a searcher for truth and facts. He presents in this edition the latest information of record, and in an attractive form. Scarcely a superfulous phrase is to be found in the book, and, moreover, it is so complete in its details as to appeal to every student of dietetics whether pupil or clinician.

